# Performance on a novel odor recognition memory test preferentially associates with specific cognitive measures and psychophysical characteristics

**DOI:** 10.1002/alz70857_106541

**Published:** 2025-12-25

**Authors:** Tiana M. Saak, Renjie Zhang, Matthew D.A. Spence, Davangere P. Devanand, Jonathan B. Overdevest, Jeffrey N. Motter

**Affiliations:** ^1^ Columbia University Vagelos College of Physicians and Surgeons, New York, NY, USA; ^2^ New York State Psychiatric Institute, New York, NY, USA; ^3^ Columbia University Irving Medical Center, New York, NY, USA; ^4^ New York‐Presbyterian Hospital, New York, NY, USA

## Abstract

**Background:**

Olfaction and memory are each independently disrupted in Alzheimer's disease (AD) early in the clinical course. Odor memory measures combining these two systems may be more capable of distinguishing these disorders than measure of either system in isolation. The purpose of this study is to evaluate psychophysical and demographic factors affecting odor memory performance and the association of odor memory performance with well‐established measures of olfaction and cognition.

**Method:**

*N* = 47 participants (Mean age=33.4, SD=14.6, range=19‐60, 48.9% female) with intact olfaction and cognition completed a battery of olfactory and cognitive tests. The novel odor recognition memory test (ORMT) had an encoding phase during which participants rated familiarity of ten odors, followed by a 20‐minute delayed yes/no recognition phase involving ten old and ten new odors. The discriminability index (d') was the primary outcome variable. Pearson correlations, t tests, and Wilcoxon tests were used to evaluate association with other measures and demographic variables.

**Result:**

Performance on the ORMT (d') was strongly correlated (r[45]=0.50, *p* <0.001) with performance on a visual recognition memory test (Rey Complex Figure Test [RCFT], d'). Importantly, ORMT performance was not significantly correlated with performance on measures of dissimilar cognitive domains, including global cognition (MoCA, r=0.05, *p* = 0.725), visuospatial constructional ability (RCFT Copy, r=0.11, *p* = 0.453), odor identification (r[45]=0.17, *p* = 0.266), odor discrimination (r[45]=0.23, *p* = 0.119), odor threshold (r[45]=0.11, *p* = 0.455), or Sniffin' Sticks TDI composite (r[45]=0.26, *p* = 0.082). Odors rated as 'familiar' during encoding were significantly more often true positives during recognition than 'unfamiliar' odors (W=825, z=‐5.12, *p* <0.001). Odor intensity and pleasantness ratings were not significantly associated with recognition. ORMT performance was not associated with age (*r* = 0.02, *p* = 0.871) or sex (t[45]=0.94, *p* = 0.353).

**Conclusion:**

In cognitively intact individuals, the ORMT exhibited preliminary evidence of convergent validity through strong association with a well‐established visual recognition memory test and evidence of divergent validity through unrelatedness to distinct cognitive and olfactory constructs. This simple, inexpensive, well‐tolerated test represents a method of assessing recognition memory using a sensory domain less prone to interference effects. Since AD biomarkers associate with odor memory measures beyond psychophysical olfaction, ongoing studies in larger populations at‐risk for AD will determine the prognostic utility of the ORMT.

